# Supplementary material for: Spatial Heterogeneity Effects on Meta-Community Stability of Annual Plants from a Coastal Dune Ecosystem
Source: Plants (Basel). 2023 May 29;12(11):2151. doi: 10.3390/plants12112151 (PMC10255161; doi:10.3390/plants12112151)
Supplement: Supplementary file 1 [file plants-12-02151-s001.zip › plants-2425532-supplementary.pdf]

Article

Spatial Heterogeneity Effects on Meta-Community Stability of Annual Plants from a Coastal Dune Ecosystem

Pua Bar (Kutiel) [1], Ofir Katz [2,3] and Michael Dorman [1]*

[1] Department of Geography and Environmental Development, Ben-Gurion University of the Negev, Be'er-Sheva 84105, Israel; kutiel@bgu.ac.il
[2] Dead Sea and Arava Science Center, Mt. Masada, Tamar Regional Council, Tamar 86910, Israel; katz.phyt@gmail.com
[3] Eilat Campus, Ben-Gurion University of the Negev, Hatmarim Blv, Eilat 8855630, Israel
* Correspondence: dorman@bgu.ac.il

Supplementary information

Species	Cover (%)
Rumex pictus	1.94269
Senecio joppensis	1.06716
Rumex bucephalophorus	0.69077
Hormuzakia aggregate	0.60344
Crepis aculeata	0.45249
Ifloga spicata	0.27869
Daucus glaber	0.27100
Polycarpon succulentum	0.20392
Lotus halophilus	0.19491
Geranium robertianum	0.12869
Corynephorus articulatus	0.09241
Maresia pulchella	0.08917
Lupinus palaestinus	0.08669
Phleum exaratum	0.08472
Trisetaria linearis	0.07674
Bromus rigidus	0.05853
Anagallis arvensis	0.05526
Torilis arvensis	0.05252
Bromus sterilis	0.04285
Lupinus angustifolius	0.04055
Brassica tournefortii	0.03556
Asphodelus tenuifolius	0.03319
Erodium laciniatum	0.03162
Euphorbia terracina	0.03155
Cutandia memphitica	0.03052
Ononis serrata	0.02360
Neurada procumbens	0.02296
Avena barbata	0.02262
Urospermum picroides	0.02245
Campanula sulphurea	0.01914
Lathyrus marmoratus	0.01835
Arenaria leptoclados	0.01613
Plantago sarcophylla	0.01599
Galium aparine	0.01528

Centaurea procurrens	0.01294
Lagurus ovatus	0.01197
Pancratium maritimum	0.01104
Vicia sativa	0.00902
Sonchus oleraceus	0.00769
Lamium amplexicaule	0.00742
Trifolium tomentosum	0.00674
Hordeum glaucum	0.00665
Hypochaeris glabra	0.00624
Stellaria media	0.00546
Astragalus boeticus	0.00513
Rumex occultans	0.00502
Maresia nana	0.00474
Crepis aspera	0.00433
Mercurialis annua	0.00418
Launaea fragilis	0.00411
Urtica urens	0.00356
Vulpia fasciculata	0.00329
Crucianella macrostachya	0.00296
Silene sp.	0.00288
Galium philistaeum	0.00261
Crucianella aegyptiaca	0.00249
Senecio leucanthemifolius	0.00232
Heterotheca subaxillaris	0.00214
Anchusa undulata	0.00190
Lactuca serriola	0.00166
Sonchus tenerrimus	0.00158
Tragopogon coelesyriacus	0.00150
Lathyrus hierosolymitanus	0.00142
Paronychia argentea	0.00142
Astragalus annularis	0.00142
Solanum villosum	0.00131
Geranium molle	0.00119
unknown	0.00119
Bromus madritensis	0.00119
Trifolium palaestinum	0.00115
Trifolium campestre	0.00107
Lolium rigidum	0.00071
Conyza sp.	0.00058
Aegilops sp.	0.00024
Brachypodium sp.	0.00024
Malva parviflora	0.00024
Astragalus palaestinus	0.00012
Vicia peregrina	0.00012
Trifolium philistaeum	0.00001
Veronica cymbalaria	0.00001

Table S1. Average cover, across all plots, of the recorded annual plant species
